# Algae extract-based nanoemulsions for photoprotection against UVB radiation: an electrical impedance spectroscopy study

**DOI:** 10.1038/s41598-025-85604-z

**Published:** 2025-01-14

**Authors:** Aura Rocío Hernández, Lady Sepulveda, Yoshie Hata, Leonardo Castellanos, Sebastian Björklund, Tautgirdas Ruzgas, Marcela Aragón

**Affiliations:** 1 Department of Biomedical Science, Faculty of Health and Society, Malmö University, 205 06 Malmö, Sweden; 2 Biofilms Research Center for Biointerfaces, Malmö University, 205 06 Malmö, Sweden; 3 Departamento de Farmacia, Facultad de Ciencias, Universidad Nacional de Colombia, Cra. 30 N° 45-03, Bogotá D.C., Colombia; 4 Departamento de Química, Facultad de Ciencias, Universidad Nacional de Colombia, Cra. 30 N° 45-03, Bogotá D.C., Colombia; 5 Instituto de Biotecnología, Facultad de Ciencias, Universidad Nacional de Colombia, Cra. 30 N° 45-03, Bogotá D.C., Colombia

**Keywords:** Nanoemulsions, Photoprotection, Electrical impedance spectroscopy, *Dictyopteris justii*, *Sargassum cymosum*, Drug delivery, Drug delivery, Marine chemistry, Electrochemistry, Skin cancer, Skin models

## Abstract

Skin cancer is one of the most common types of cancer worldwide, with exposure to UVB radiation being a significant risk factor for its development. To prevent skin cancer, continuous research efforts have focused on finding suitable photoprotective ingredients from natural sources that are also environmentally friendly. This study aimed to develop oil-in-water photoprotective nanoemulsions containing marine macroalgae extract. A Box–Behnken experimental design was used to identify the most promising formulation composition, resulting in optimal physical properties. These properties, including droplet size, polydispersity index (PDI), and zeta potential, were evaluated using dynamic light scattering (DLS). To assess the photoprotection capacity of the formulations, electrical impedance spectroscopy (EIS) was employed to evaluate alterations in the electrical characteristics of excised pig skin membranes placed in Franz cells equipped with a 4-electrode set-up. The final composition of the nanoemulsion was caprylic/capric triglycerides 4%, Macrogolglycerol ricinoleate 30%, and algae extract 1%. The nanoemulsions had an average droplet size of 128.5 ± 8.6 nm, a PDI of 0.25 ± 0.06, and a zeta potential of 45.14 ± 0.02 mV. Compared to the control group, the photoprotective capacity of the oil-in-water nanoemulsions was statistically significant. Specifically, only a 15% reduction in the skin membrane electrical resistance following UVB exposure was observed when the formulation containing algae extract was used, whereas a 50% reduction was observed for the vehicle. In conclusion, this work demonstrates that the developed nanoemulsions based on natural ingredients show promising protective capacity against UVB exposure of the skin.

## Introduction

The skin is the body’s largest organ and performs many important functions, including acting as a barrier against water loss and providing protection against xenobiotics^1,2^. It has multiple defense mechanisms that support the maintenance of homeostasis^3^. Despite these defenses, exposure to ultraviolet (UV) radiation and the resulting oxidative stress^4^ pose significant challenges to skin health and are recognized as major causes of various skin diseases^5^.

The sun is the primary source of UV radiation, and excessive and uncontrolled UV exposure can cause irreversible harm, including DNA damage leading to skin cancer, deactivation of antioxidative enzymes, increased oxidative stress, alteration of the molecular mobility of lipids and proteins in the stratum corneum (SC), and compromised SC mechanical integrity^6–9^. Nevertheless, solar radiation also has several beneficial effects. For example, UV radiation is essential for vitamin D3 production in the epidermis and dermis, and there is growing evidence of its positive impact on systemic autoimmune diseases such as multiple sclerosis, noncancer mortality, and the prevention of myopia^10,11^.

In recent years, natural ingredients have drawn considerable attention because of their inherent developed capability to protect against the deleterious effects of oxidative stress and UV radiation, which can be explained by their antioxidant activity and capacity to absorb harmful UV rays^12,13^. Marine algae extracts, which are rich in antioxidants, vitamins, and pigments^14^, offer a sustainable and environmentally friendly option for sun protection^15^. Brown macroalgae, which are widely distributed on the Caribbean coast, contain many phenolic compounds, such as catechins, phlorotannins (unique to brown algae), flavonoids and flavanols. Owing to these compounds, brown macroalgae can be used diversely because of their potent antioxidant, antiaging, and antimicrobial activities^16,17^, as well as the remarkable photoprotective activity reported for compounds such as fucoidans^18,19^ and fucoxanthin^20,21^. Mycosporine-like amino acids (MAAs), which are predominantly found in reddish algae, have demonstrated remarkable photoprotective and antioxidant properties^22^. One of the most promising compounds from MAAs is Porphyra-334, which has already been registered as a natural cosmetic ingredient due to its photophysical and photochemical properties^23,24^.

The evaluation of photoprotective activity is crucial for sunscreen development and there is a need for more reliable, reproducible, and accurately predictive alternatives. Traditional methods rely on in vivo testing through the determination of sun protection factor (SPF). SPF is a value that determines the ability of a sunscreen product to prevent sunburn caused by solar radiation. It is calculated in vivo as the ratio between the minimum dose of UVB radiation required to produce erythema on the skin with the product of interest and the minimum dose of UVB radiation required to produce erythema on unprotected skin^25^. The SPF can also be related to the amount of energy in photons that is absorbed or filtered by the sunscreen compared with the amount of UV rays that reach the skin. In theory, an SPF 10 sunscreen should only allow 1/10th of the UV rays to reach the skin, blocking 90% of the radiation. Similarly, an SPF 50 sunscreen permits merely 1/50th of the UV rays to reach the skin in theory, whereas 98% of the radiation is blocked^26,27^. Regarding the determination of the SPF value of a sun protectant, it must be made for each new formulation intended for commercialization and is standardized under ISO 24444:2010. To date, no alternative methodology has been accepted by global regulatory agencies to replace this approach. Many in vitro methods struggle to fully replicate the complexity of skin responses under real-world conditions, including oxidative stress, long-term UV exposure, and environmental factors. As a result, research in this area continues to develop alternative methods that allow the replacement of human testing due to the ethical questions it raises while also closely approximates the biological response for better prediction and facilitating the evaluation of these products for more timely market entry^28,29^.

The cosmetic industry is one of the most dynamic and rapidly evolving sectors globally, with new formulations being launched constantly to improve product functionality and quality^30^. Notably, there is a trend toward optimizing vehicles for natural compounds^31,32^. Given that traditional SPF determination is time-consuming and resource-intensive, simple yet realistic in vitro methodologies can provide valuable insights at minimal cost. This is particularly relevant in the early stages of research and development, where new photoprotective agents in combination with various excipients of sun-protective formulations require simple and fast in vitro characterization. At present, common in vitro methods are based on UV spectrometry, where UV absorption or transmission is determined by applying the product either onto transparent solid substrates, such as quartz^33^, polymethyl methacrylate^34,35^, or biomembranes^36,37^, or by simply measuring the UV absorption characteristics in diluted solutions^38–40^. Additionally, some studies have reported the use of electrical impedance to demonstrate changes in skin integrity both in vitro^41–43^ and in vivo^44^. In the field of sun protection, electrical impedance spectroscopy (EIS) and DNA electrochemical sensors have also been employed as alternatives for evaluating sunscreen agents^45^. In our previous work, we demonstrated that EIS is suitable for studying the effects of UVB irradiation on the skin in the presence of oxidative stress. Additionally, the EIS methodology successfully allows the evaluation of the protective capacity of topical sunscreen formulations with different SPFs, which is strongly correlated with the electrical skin membrane resistance^46^.

Considering that nanoemulsions represent promising carriers of various ingredients with beneficial photoprotection properties^47–49^, the first aim of this study was to develop oil-in-water (o/w) nanoemulsions with incorporated extracts from two brown algae. The selected algae were *Dictyopteris justii* and *Sargassum cymosum*, both of which potentially contain extractable substances with photoprotective properties. Nanoemulsions are kinetically stable colloidal dispersions consisting of oil, water, and surfactants (sometimes with co-surfactants), containing nano-sized droplets produced through high-energy or low-energy methods^50^. In cosmetic formulations, oil-in-water nanoemulsions are commonly preferred due to their small droplet size, large surface area, low viscosity, transparent or translucent appearance, high solubilization capacity, reduced surfactant requirements, and ability to protect encapsulated active compounds^51^. To optimize the nanoemulsions, a Box–Behnken experimental design, relying on response surface methodology, was employed. The advantage of using this statistical approach is primarily the ability to evaluate the effects and interactions of multiple independent variables with fewer experimental runs compared to traditional methods. In particular, the Box–Behnken design is helpful for achieving precise control of parameters influencing nanoemulsion properties, such as droplet size, polydispersity index, and zeta potential, which are pivotal for stability and efficacy^52–54^. The second goal was to evaluate the protection capacity of the optimized nanoemulsions against damaging effects due to UVB exposure, which was achieved by probing changes of the electrical properties of excised skin membranes in vitro using EIS.

In summary, this study demonstrates a promising method of integrating photoprotective substances derived from marine algae into nanoemulsion-based formulations. We show that the photoprotective efficacy of these formulations can be assessed using the relatively simple, efficient, and reliable in vitro EIS methodology. This technique evaluates the formulation’s ability to maintain skin membrane integrity against the harmful effects of UVB radiation and oxidative stress, which would otherwise significantly reduce skin membrane resistance, indicating a compromised skin barrier.

## Materials and methods

### Materials

Methanol (MeOH), dichloromethane (DCM) and deuterated chloroform (CDCl_3_) were purchased from Merck KGaA. Caprylic/capric triglycerides (Kollisolv® MCT 70) and macrogolglycerol ricinoleate (Kolliphor® EL) were purchased from BASF GmbH (Germany). Butyl hydroxytoluene (BHT), propylene glycol, methylparaben and propylparaben (MP and PP), tablets for phosphate-buffered saline (PBS, pH 7.4), sodium azide (NaN_3_) and hydrogen peroxide (H_2_O_2_, 30%, 9.8 M) were purchased from Sigma Aldrich (Germany). All the solutions were prepared from deionized water with a resistivity of 18.2 Ω cm. Commercial sunscreen with SPF 100 containing both organic and inorganic UV filters was purchased from the market and used as a reference for photoprotection.

The brown macroalgae *Dictyopteris justii* YP17501 (YP) was collected at Providencia Island, Colombia, in July 2021, and *Sargassum cymosum* GM1039 (GM) was collected at Santa Marta, Colombia, in April 2022. The samples were kept at − 20 °C, and the identification process was conducted by Dr. Briggite Gavio and Dr. Vanessa Urrea-Victoria based on morphological characteristics using descriptions from the relevant literature^55,56^. The algal nomenclature and taxonomy followed AlgaeBase^57^. Individual specimens of each sample were deposited at the JIWUKORI Algae Herbarium at Universidad Nacional de Colombia. The samples were washed with artificial seawater to eliminate debris and epiphytes, followed by lyophilization for 24–48 h until complete dryness was observed.

### Methods

#### Algae extraction

Briefly, 22 g dry weight of each genre was extracted with 440 mL of MeOH/water (1:1), and the aqueous crude extract (A) was obtained. The remaining algae material was further extracted with 440 mL of DCM/MeOH (1:1), yielding the organic extract used in our study. Each extraction was performed with ultrasonic assistance for 1 h. The supernatants were recovered by filtration, dried under vacuum, and stored in darkness at − 20 °C. Following this procedure, 473 mg of organic extract of *D. justii* and 219 mg of organic extract of *S. cymosum* were obtained^58^.

#### ^1^H nuclear magnetic resonance (NMR) spectroscopy of algae extracts

The extracts were dissolved in an appropriate deuterated solvent (CDCl₃). A Bruker AVANCE NMR spectrometer operating at 400.13 MHz and 295.8 K was used. Each ^1^H NMR spectrum was acquired with a width of 10 ppm, 16 scans and a relaxation delay of 1.0 s. The residual H_2_O signal was suppressed using NOESYgppr1d. The structural characterization was established through the analysis and interpretation of the chemical shifts (δ, in ppm) in the 1H NMR spectrum studies and the correlations obtained from two-dimensional NMR studies: ^1^H-^1^H COSY, HSQC (NUS 128 Scans), and HMBC^59^. The metabolites were identified by comparing the NMR spectral data with those in the literature.

#### UHPLC-DAD analysis of the algae extracts

A Thermo Scientific Dionex Ultimate 3000 chromatograph equipped with a Dionex Ultimate 3000 diode array detector (DAD), Dionex Ultimate 3000 RS quaternary pump, in-line degasser, and automatic injector was used. A Phenomenex Kinetex C8 column (150 mm × 4.6 mm, 5 μm) was used at 30 °C. A mixture of water (A) and acetonitrile (B) was used to create a 20 min gradient elution as follows: 60% solvent B was maintained for 2 min, then increased to 95% from 2 to 18 min and held for 2 min at a flow rate of 0.8 mL/min. The detection was performed at a wavelength of 470 nm, and 25 μL of sample was injected^60^.

### Cytotoxicity assay of algae extracts

Potential toxic effects on skin from the algae extracts were investigated via a mitochondrial activity test (i.e., MTT assay) based on the conversion of 3-[4,5-dimethylthiazol-2-yl]-2,5 diphenyl tetrazolium bromide into formazan crystals by living cells. Fibroblasts (NIH/3T3) from mouse NIH/Swiss embryos (ATCC CRL-1658) were obtained from the American Type Culture Collection (ATCC) (Manassas, VA, USA). They were grown in DMEM supplemented with 10% FBS and antibiotics (60 mg/L gentamicin) in a 5% CO_2_ atmosphere at 37 °C. A trypsin–EDTA solution was used to detach the cells. The cells were seeded in a 96-well plate with 10 × 10^4^ cells in 100 µL in each well. After 24 h, the cells were treated with each algae extract for 24 h at concentrations of 25, 50, 100, 200, and 400 μg/mL, which were previously dispersed in DMSO. As a negative control, the cells were treated with culture medium and DMEM without FBS (i.e., untreated cells). As a positive control (i.e., a toxic agent), the cells were treated with serial dilutions of 50 mM etoposide in DMEM without FBS^61^. The MTT reagent was incubated with a final concentration of 1 mg/mL for 4 h at 37 °C. The MTT solution was removed carefully, and 100 μL of DMSO was added to solubilize the formazan crystals. The absorbance was measured at a wavelength of 570 nm using a TRIAD Multimode Microplate Reader (DYNEX Technologies, Inc., Chantilly, VA, USA). The absorbance interference from the cells was removed by subtracting the absorbance from that of the untreated cells. The percentage of viable cells was calculated as the ratio of the average absorbance of treated cells to that of untreated cells. For the calculation of the parameter IC_50_, we used cell viability (%) versus concentration (mg extract/mL).

### Formulation of nanoemulsions containing* algae extracts*

Nanoemulsions consisting of water, oil (caprylic/capric triglycerides), and a surfactant (macrogolglycerol ricinoleate) were prepared using an ultrasonicator (Q500-SONICATOR, Newton, MA, USA) with 80% amplitude for 4 min. To identify the nanoemulsion region, a ternary phase diagram was constructed based on 24 different compositions, where the contents of oil and surfactant varied between 4 and 20%. All formulations were characterized after 1 and 10 days of preparation by dynamic light scattering (DLS) using a Zetasizer nano-ZS (dispersion angle 173°, 25 °C) (Worcestershire, UK). For determine stability of different systems i.e., after 10 days, samples were stored at 20 °C under 12-h daily light exposure. For DLS measurements, 10 µL sample aliquots were diluted in 1 mL of deionized water and analyzed in terms of the mean droplet size, polydispersity index (PDI), and zeta potential (ζ)^62^. The statistical analysis was performed using Student´s t test with the Excel data analysis add-on XLSTAT 2018.1.

### Experimental design for nanoemulsions containing algae extracts

To find the optimal emulsification conditions needed to obtain the most suitable formulation with the algae extract, considering the lowest droplet size and PDI value, a Box–Behnken design (BBD) was created using the statistical software program Minitab 18 (Copyright 2018. Minitab Inc., State College, PA, USA). The design considered three factors (oil %, surfactant %, and emulsification time) with three levels each (high, medium, and low) based on the results obtained previously in the ternary phase diagram. The response variable chosen was droplet size, although the PDI and zeta potential were also monitored. A total of 15 samples for each extract were evaluated according to the BBD^63^. The collected data for the variables were analyzed using the response surface methodology (RSM) within the same software.

### Preparation of skin membranes

Fresh pig ears were obtained from a local abattoir and used to prepare skin membranes according to the following methods. First, the fresh ears were rinsed with cold water and cut into strips with a scalpel. Hair was removed by an electrical clipper. The skin from the inner part of the ears was subsequently extracted from the tissue strips using a dermatome (TCM 3000 BL, Nouvag AG, Goldach, Switzerland), resulting in skin pieces approximately 0.5 mm thick. Circular membranes with a 16 mm diameter were then punched out from these skin pieces to fit the Franz cell, which was utilized for impedance measurements. The membranes not immediately utilized were stored at − 20 °C on filter paper soaked in PBS and used within two weeks^64^.

### UVB irradiation and dosage

A Thermo Scientific™ 3UV lamp (230 V, 50 Hz) was used as the source of radiation, with a narrowband UVB bulb at 302 nm. Stable radiation was obtained after the lamp was preheated for 10 min. Rutinary controls were used to confirm the irradiation output using a UV meter (UV-340A, Lutron Electronic Company, Taipei, Taiwan). The radiance was determined to be 0.01 W/cm^2^ at 2 cm, corresponding to the gap between the skin membrane and the light source. With this setup, an exposure time of 5 h was used to obtain a UVB dose of 180 J/cm^2^. Notably, this UVB dose is equivalent to approximately two weeks of solar UVB exposure^9^ and significantly surpasses the minimal erythema dose (MED) that causes skin reddening and inflammation^5^. This high UVB dose was not chosen to mimic specific real-world conditions but rather to systematically investigate the potential of algae-based nanoemulsions to protect the skin barrier from the damaging effects of UVB radiation.

### Evaluation of the photoprotective activity of the formulations using electrical impedance spectroscopy (EIS)

**Fig. 1:**
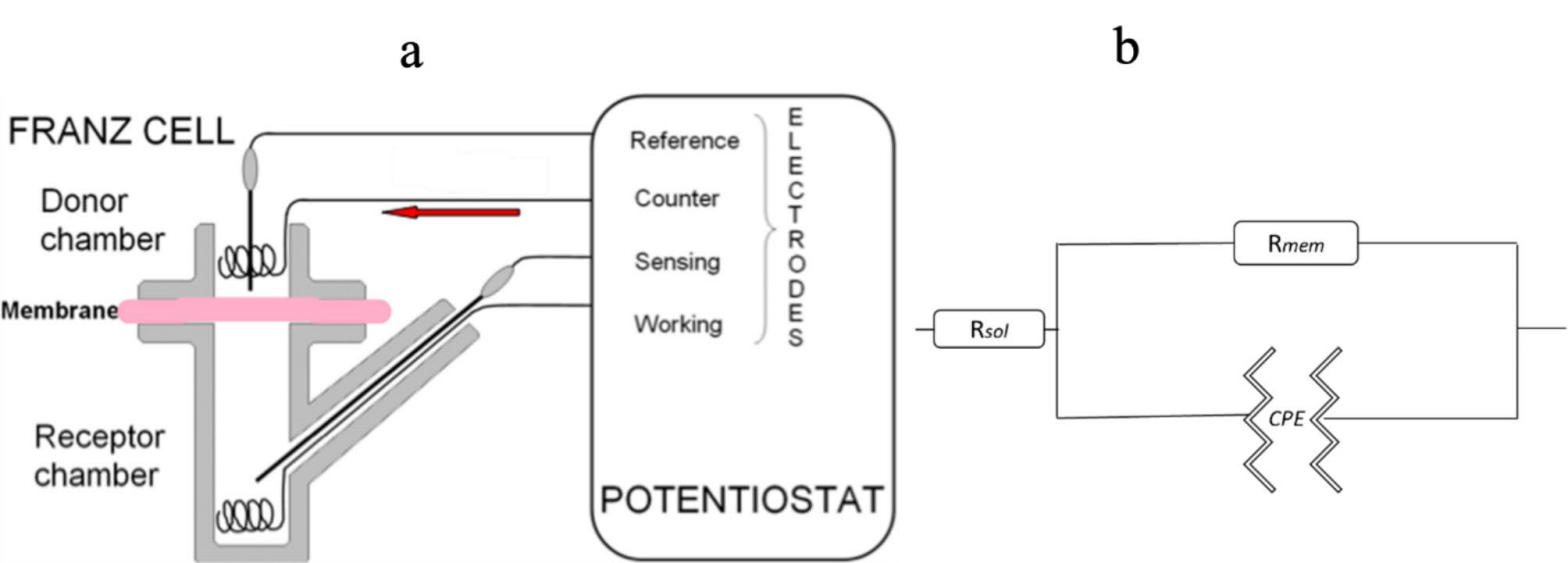
(**a**) Schematic diagram of the 4-electrode setup: two platinum wires functioned as working and counter electrodes, while two Ag/AgCl/3 M KCl electrodes were used as sensing and reference electrodes; (**b**) Equivalent circuit model of the skin membrane, where *R*_sol_ represents the resistance of the donor and receptor solutions, R_*mem*_ is the membrane resistance, and *CPE* is a constant-phase element used to determine the effective capacitance of the membrane *C*_eff_.

EIS measurements were performed as previously reported^46^ using a Franz cell (Ø = 0.90 cm, V = 6 mL, PermeGear Inc.) with a four-electrode configuration shown in Fig. 1a. The electrodes were connected to an Ivium Technologies potentiostat. Platinum wires served as the working and counter electrodes, whereas Ag/AgCl/3 M KCl electrodes (World Precision Instruments Germany GmbH. Friedberg, Germany) were used as the sensing and reference electrodes. All the measurements were conducted at room temperature, encompassing a frequency range from 0.1 Hz to 1 MHz, with an applied voltage amplitude of 100 mV. In its most basic form, impedance represents the relationship between voltage and current across a range of frequencies. The measurement involves applying an alternating sinusoidal potential (voltage) between the working and counter electrodes, ensuring that the potential difference between the working and reference electrodes matches the set value of the potentiostat. The applied potential difference induces a response current between the counter and working electrodes, which is determined by the potentiostat. The impedance characteristics of the skin membrane include both resistive and capacitive elements. In this study, EIS data were analyzed using a skin equivalent circuit model comprising a resistor (representing solution resistance, *R*_sol_) in series with a parallel combination of a resistor (representing skin membrane resistance, *R*_mem_) and a constant phase element (*CPE*, representing the heterogeneous capacitor of the skin), as depicted in Fig. 1b. In accordance with our previous findings, the resistance of the skin membrane was used as a response variable derived from the real part of the impedance in the frequency regions where the imaginary part minimally contributes to the total impedance. For this analysis, all the data were normalized to the skin membrane area (0.64 cm^2^) to obtain units in Ohm·cm^2^. The effective capacitance *C*_eff_ was calculated from the high-frequency region from the imaginary impedance data by a procedure described in detail in a previous study^65^. The EIS experiments were designed to minimize the natural variability by analyzing impedance data from individual membranes base on the changes in *R*_mem_ and *C*_eff_ over time (t) relative to their initial (i) values, as described in Eqs. (1) and (2):

1 $${\Delta R}_{mem}=\frac{{R}_{mem, t}-{R}_{mem, i}}{{R}_{mem, i}}\times 100\%$$

2 $${\Delta C}_{eff}=\frac{{C}_{eff, t}-{C}_{eff, i}}{{C}_{eff, i}}\times 100\%$$

The photoprotective capacity of nanoemulsions was investigated by EIS following a previously published procedure^46^. In brief, oxidative stress conditions were induced by exposing the skin membrane to PBS containing 1 mM H_2_O_2_ and 10 mM NaN_3_ in both the donor and receptor compartments of the Franz cell. Initial EIS measurements were performed over 3 h in PBS to establish reference values of ∆R_mem_ and ∆C_eff_. Following this, the formulations were topically applied using a standard dose of sunscreen formulation (2 mg/cm^2^), followed by UVB exposure for 5 h in total (corresponding to a dosage of 180 J/cm^2^). Using this protocol, the effect of UVB exposure on the electrical properties of excised skin membranes was evaluated by the following experimental conditions:

- Without any topically applied formulation (i.e., SPF 0)
- Topically applied nanoemulsion with macroalgae extract GM
- Topically applied nanoemulsion with macroalgae extract YP
- Topically applied nanoemulsion vehicle (without any macroalgae extract)
- Topically applied commercial sunscreen formulation with SPF 100

In all the cases, 6 skin membranes were used for each type of treatment. The UVB exposure was performed in a closed chamber ensuring high humidity (i.e., avoiding dryness). In addition, control experiments without exposure to UVB were performed for each experimental condition and included as a reference in the results.

### Statistical analysis

To evaluate if the observed changes in the mean values of ∆R_mem_ and ∆C_eff_ were significantly different among the five groups, we performed a one-way ANOVA, assuming equal variances. Following the ANOVA, a multiple comparison test (Tukey test) was conducted to identify which specific groups differed and the significance of these differences^66^. The analyses were performed using GraphPad Prism Software 10.2.2.

## Results and discussion

### NMR analysis of algae extracts

**Fig. 2:**
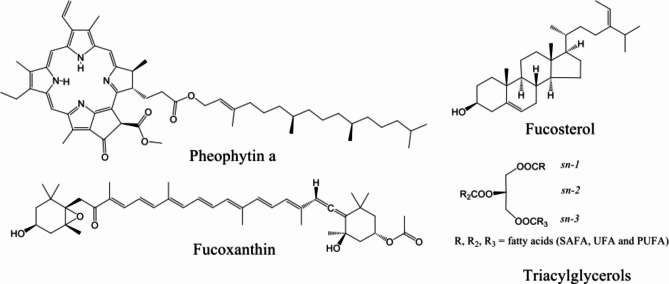
Chemical structures of the main compounds identified in both the *S. cymosum* (GM) and *D. justii* (YP) extracts. Pheophytin a, fucosterol and triacylglycerols were identified by NMR. Fucoxanthin was identified by HPLC–DAD. Chemical structures were created by ChemDraw.

The algal extracts were analyzed by NMR. Abundant compounds were identified by dereplication procedures using data reports from scientific articles^67^. The most abundant compounds for the *S. cymosum* (GM) and *D. justii* organic extracts corresponded to fatty acids SAFA (saturated fatty acids), UFA (unsaturated fatty acids), PUFA (polyunsaturated fatty acids) and their derivatives, such as triacylglycerols, as shown in Table 1. The cross peaks in the HMBC and COSY spectra confirmed the compounds presented in Fig. 2 and that have been reported by other authors^68^.

**Table 1:** Characteristic ^1^H NMR signals of compounds identified in the algal extracts of *S. cymosum* (GM) and *D. justii* (YP) (s: singlet; d: doublet; *J*: coupling constant).

Compound	Position	δ_H_	δ_C_
Triacylglycerols	*sn2*	4.32	62.3
		4.19	
	*sn1,3*	5.29	67.7
SAFA	CH_3_	0.91	14.0
	–(CH_2_)_n_–	1.35–1.19	30–22
UFA	CH_3_ ω-9	0.91	14.0
	CH_3_ ω-6		
	CH_3_ ω-3	0.99	14.1
	–(CH_2_)_n_-	1.35–1.19	30–22
	CH_2_ α to C=O	2.34	34.3
	allylic CH_2_	2.04	27.2
PUFA	Bisallylic CH_2_	2.84	25.7
	Olefinic protons	5.37	129.8
		5.38	128.3
*Pheophytin a*	α	9.40	97.6
	β	9.54	104.5
	δ	8.58	93.1
	1a	3.42	12.1
	2a	7.99	129.2
	2b-cis	6.20	122.8
	2b-trans	6.32	122.8
	3a	3.25	11.2
	4a	3.9	52.2
	4b	δ_H-4b_ 1.71	δ_C-4b_ 17.4
	5a	δ_H-5a_ 3.71	δ_C-5a_ 12.2
	7	δ_H-7_ 4.23	δ_C-7_ 51.1
	8	δ_H-8_ 4.48	δ_C-8_ 50.2
	8a	δ_H-8a_ 1.83	δ_C-8a_ 23.1
	10	δ_H-10_ 6.29	δ_C-10_ 64.7
	10b	δ_H-10b_ 3.91	δ_C-10b_ 52.8
	P4	δ_H-P4_ 1.91	δ_C-P.4_ 39.9
	P11a/P15a/P16a	δ_H-P11a/P15a/P16a_ 0.87	δ_C-P11a/P15a/P16a_ 22.6
Fucosterol	CH_3_-18	δ_H-18_ 0.70 (s)	δ_C-18_ 11.8
	CH_3_-26/CH_3_-27	δ_H-26/27_ 0.97 (d, *J* = 6.8)	δ_C-26/27_ 22.3
	CH_3_-21	δ_H-21_1.00 (d, *J* = 6.8)	δ_C-21_19.1
	CH_3_-19	δ_H-19_ 1.02 (s)	δ_C-19_19.1
	CH_3_-29	δ_H-29_ 1.59 (d, *J* = 7.7)	δ_C-29_13.3
	CH-28	δ_H-28_ 5.16	δ_C-28_ 117.8
	CH-6	δ_H-6_ 5.36	δ_C-6_ 121.7

### HPLC‒DAD analysis of fucoxanthin

**Fig. 3:**
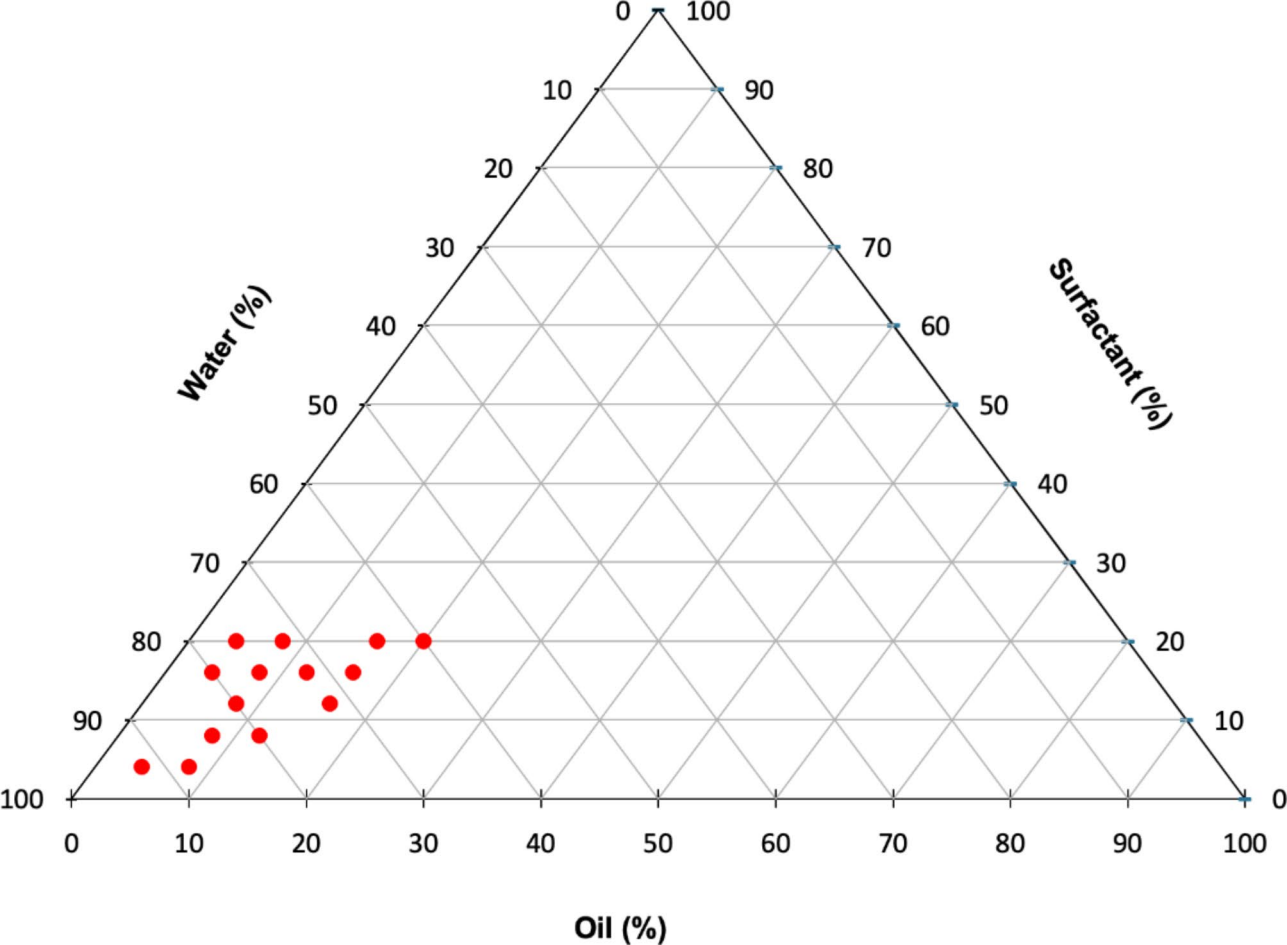
Ternary diagram for the determination of the nanoemulsion region. Oil: caprylic/capric triglycerides; surfactant: macrogolglycerol ricinoleate.

The algal extracts were analyzed by HPLC–DAD, where an organic extract of *Undaria pinnatifida*, which is rich in fucoxanthin, was used as a reference. In general, the UV spectra of the crude extracts presented carotenoid absorption characteristics (350–530 nm). The chromatogram shown in Fig. 3 displays a large peak at 13.8 min, with UV maxima at 445 and 470 nm. The same peak was observed in a semipurified fraction of the GM extract, with the same retention time. In the case of the analogous fraction of the YP extract, the chromatogram has this peak, but it is smaller, in addition to other peaks at 17.0, 17.5, and 20.0 min, which are characteristic of the UV shape of carotenoids and may correspond to lutein derivatives according to a previous report^69^. In conclusion, both extracts contain fucoxanthin, with the GM extract having a greater amount than the YP extract, which also contains other carotenoids.

### Safety assessment of algae extracts

To investigate the safety profiles of the extracts, toxicity was assessed by MTT assay, which is broadly used to measure the in vitro cytotoxic effects of different compounds on cell lines. In this case, the fibroblasts survived after the treatments were applied. Thus, we demonstrated that both the *S. cymosum* and *D. justii* extracts were noncytotoxic at the different doses used. After 24 h of incubation, the cell viability was maintained at the highest concentration used for the extracts with an IC_50_ > 1000 µg/mL, i.e., no toxic effect on fibroblasts was detected (no significant differences vs. the control, α = 0.05). These results indicate that the extracts are safe for use in topical formulations and support the findings of previous studies investigating the cytotoxicity of red algae extracts^22^.

### Formulation of the nanoemulsion

#### Construction of the ternary diagram

Twenty-five nanoemulsions were prepared by varying the proportions of the dispersed phase, dispersing phase, and surfactant, with the aim of evaluating the influence of composition on the formation of nanoemulsified systems. The variation in the proportions of the emulsions was defined based on previous studies^62^, with percentages of oil ranging from 4 to 20%. Sonication, which is a high-energy method suitable for generating emulsions with small droplet sizes, was chosen as the preparation method. The droplet size (nm) and PDI obtained for each emulsion are shown in Table 2. Those parameters were evaluated on day 1 and day 10 in order to determine the stability of each system.

**Table 2:** Composition and droplet size of prepared nanoemulsions.

N°	Composition (%)			Day 1		Day 10	
	Oil	Surfactant	Water	Droplet Size (nm)	PDI	Droplet Size (nm)	PDI
1	4	4	92	86.5	0.254	96.0	0.207
2	4	8	88	41.8	0.360	46,0	0.372
3	4	12	84	25.3	0.175	36.7	0.340
4	4	16	80	24.9	0.238	23.0	0.167
5	4	20	76	21.1	0.189	20.0	0.112
6	8	4	88	135.4	0.163	141.9	0.124
7	8	8	84	99.4	0.217	112.0	0.184
8	8	12	80	75.7	0.262	98.70	0.240
9	8	16	76	30,0	0.058	33.5	0.124
10	8	20	72	27,7	0.164	31.3	0.193
11	12	4	84	165.7	0.143	186.5	0.204
12	12	8	80	140.1	0.261	136.4	0.173
13	12	12	76	99.3	0.298	156.7	0.354
14	12	16	72	39.6	0.147	46.3	0.133
15	12	20	68	46.5	0.353	77.3	0.435
16	16	4	80	173.2	0.127	180.5	0.095
17	16	8	76	154.9	0.153	159.2	0.131
18	16	12	72	147.1	0.233	149.4	0.112
19	16	16	68	84.3	0.309	99.2	0.267
20	16	20	64	46.4	0.126	59.6	0.137
21	20	4	76	340.4	0.390	318.1	0.379
22	20	8	72	174.0	0.172	195.9	0.189
23	20	12	68	149.9	0.161	159.7	0.144
24	20	16	64	150.1	0.184	179.5	0.195
25	20	20	60	87,8	0.188	126.3	0.187

The data are presented as the mean of three samples. The standard deviation in all the cases was < 2%

The ingredients for nanoemulsions were selected based on sustainable and eco-friendly criteria for new cosmetics. Caprylic/capric is a mixture of triglycerides of medium chain from vegetable resources and is considered a biodegradable solvent^70^. Macrogolglycerol ricinoleate, derived from castor oil, is a nonionic emulsifier with ethoxylated and branched alkyl chains. Owing to its structure, this ingredient is expected to be a suitable emulsifier for the mixing of compounds in algae extracts. Both ingredients are appropriate for use in topical skin products because of their low toxicity according to the safety data sheet provided by the manufacturer, which is publicly accessible at any time on their official website. Furthermore, formulations based on these components exhibit greater stability and are less affected by changes in pH and ionic strength compared to ionic emulsifiers^71,72^.

All the emulsions had a droplet size less than 300 nm and a PDI lower than 0.400. However, only those with a droplet size lower than 150 nm and a PDI less than or equal to 0.300 after day 10 were included in the ternary phase diagram in Fig. 3, which shows a nanoemulsion zone with an oil phase percentage between 4 and 16%, a surfactant of 4 and 20%, and an aqueous phase of 60 and 92%. These results are similar to previous findings, where the nanoemulsion zone was observed with the same amount of oil and water but with more surfactant. With macrogolglycerol ricinoleate as an emulsifier, the surfactant concentration could be lowered to merely 4% to achieve a stable nanoemulsion region, in contrast to previous studies that required 8% surfactant to obtain the same region^62,73,74^.

#### Box‒Behnken analysis

In this study, a Box–Behnken experimental design (BBD) was employed with the aim of determining the effects of the percentage of oil, surfactant, and sonication time on the droplet size, PDI, and zeta potential of each algae extract (YP and GM). However, only the droplet size was considered for optimizing the formulation since it influences the stability of the nanoemulsion and allows the effective transport of active ingredients to the skin. Based on the ternary diagram, 3 different percentages of oil (4, 8, and 12%) and surfactant (10, 15, and 20%) were evaluated, as shown in Table 3. Considering that shear force has been reported as one of the main factors influencing droplet formation, three sonication durations (2, 3.5, and 5 min) were evaluated based on previous literature^75^. Additionally, BBD included 3 center points (conditions repeated) to estimate experimental error and enhance the robustness of the model (Table 3).

**Table 3:** Box–Behnken experimental design matrix and average results for droplet size, PDI and ζ.

Experiment	Independent factors			YP17501 Extract			GM1039 Extract		
	oil (%)	Surfactant (%)	Time (min)	Droplet size (nm)	PDI	ζ (mV)	Droplet size (nm)	PDI	ζ (mV)
1	12.0	15.0	5.0	31.4	0.437	− 35.9	73.3	0.636	− 43.2
2	8.0	10.0	2.0	42.2	0.579	− 39.1	123.1	0.367	− 46.3
3	4.0	10.0	3.5	32.7	0.534	− 39.5	130.6	0.395	− 43.8
4	4.0	20.0	3.5	17.9	0.660	− 38.8	15.6	0.697	− 37.8
5	8.0	10.0	5.0	142.2	0.211	− 48.0	32.7	0.534	− 39.5
6	8.0	20.0	2.0	42.1	0.328	− 38.5	23.8	0.484	− 41.2
7	8.0	15.0	3.5	42.7	0.485	− 41.5	42.7	0.485	− 41.5
8	12.0	15.0	2.0	130.6	0.395	− 43.8	298.5	0.503	− 44.4
9	8.0	15.0	3.5	73.3	0.635	− 43.2	42.1	0.328	− 38.5
10	8.0	20.0	5.0	15.6	0.697	− 37.8	31.4	0.437	− 35.9
11	8.0	15.0	3.5	26.4	0.515	− 36.1	42.2	0.579	− 39.1
12	12.0	10.0	3.5	265.1	0.503	− 44.4	142.2	0.211	− 48.0
13	4.0	15.0	5.0	139.0	0.480	− 37.1	17.9	0.660	− 38.8
14	4.0	15.0	2.0	123.1	0.367	− 46.3	26.4	0.515	− 36.1
15	12.0	20.0	3.5	23.8	0.484	− 41.2	145.7	0.480	− 37.1

YP: *D. justii*, GM: *S. cymosum*. The data are presented as a mean of three samples. The standard deviation for droplet size and PDI in all cases was < 5%; for ζ, the standard deviation was < 2%

Accordingly, analysis of variance (ANOVA) enabled the relationships of statistical significance (*p* value < 0.05) to be established for all the models evaluated. The degree of fitness of the model was evaluated by coefficients of determination (R^2^), adjusted coefficients of determination (R^2^ adj) and the *p* value of the *lack-of-fit* test. The statistical analysis of droplet size for both emulsions containing either YP or GM algae extracts is summarized in Table 4. The results showed that the data fit a linear model with high significance (*p* < 0.002). Consequently, the *lack-of-fit* value was not statistically significant (*p* > 0.05), confirming the fit. The R^2^ values were > 0.97 for both cases, indicating that the model has a high degree of fitness with respect to droplet size variability, i.e., a more precise understanding of how factors influence the response, leading to better predictions. The R^2^ adj values decreased but were still above 0.90, indicating a relatively good prediction of the incidence of the independent variables on the droplet size. With respect to the influence of each variable and the interaction between variables, the model revealed that the percentages of oil and surfactant were significant factors (*p* < 0.05), and the only significant interaction was that between oil and surfactant (*p* < 0.05). Therefore, the following polynomial models were generated to predict the droplet size of the formulations:

**Table 4:** Analysis of variance (ANOVA) for Box–Behnken design to determine the droplet size for each extract formulation.

Droplet size analysis		*p*-value	
Variable		YP	GM
Linear terms	Oil	0.001	0.015
	surfactant	0.002	0.001
	Time	0.457	0.390
Quadratic terms	Oil^2^	0.090	0.195
	Surfactant^2^	0.112	0.040
	Time^2^	0.841	0.182
Interaction terms	Oil*surfactant	0.006	0.024
	Oil*time	0.819	0.115
	Surfactant*time	0.336	0.952
Lack of fit		0.080	0.011

Coefficient of determination	YP	GM	
R^2^	0.9769	0.9703	
R^2^ adj	0.9364	0.9108	

YP*: D. justii*, GM: *S. cymosum*. In the model, values less of *p* (< 0.05) are considered significant.

For YP17501

3 $$\begin{aligned} {\text{Y }} & = \, - {3}.{7}0 \, + { 28}.{71}\;oil - { 1}.{73}\;surfactant{-}{ 13}.{2}0\;time{-} \, 0.{38}\;oil^{2} \\ & \quad + \, 0.{19}\;surfactant^{2} + { 4}.{43}\;time^{2} {-} \, 0.{55}\;oil \, * \, surfactant \\ & \quad {-} \, 0.{22}\;oil \, * \, time + \, 0.{61}\;surfactant \, * \, time. \\ \end{aligned}$$

For GM1039

4 $$\begin{aligned} {\text{Y }} & = { 619 }{-}{ 8}.{36}\;oil{-}{ 36}.{58}\;surfactant{-}{ 15}.{6}0\;time + \, 0.{34}\;oil^{2} \\ & \quad + \, 0.{67}\;surfactant^{2} + { 5}.{58}\;time^{2} + \, 0.{44}\;oil \, * \, surfactant \\ & \quad + \, 0.0{4}\;surfactant \, * \, time. \\ \end{aligned}$$

**Fig. 4:**
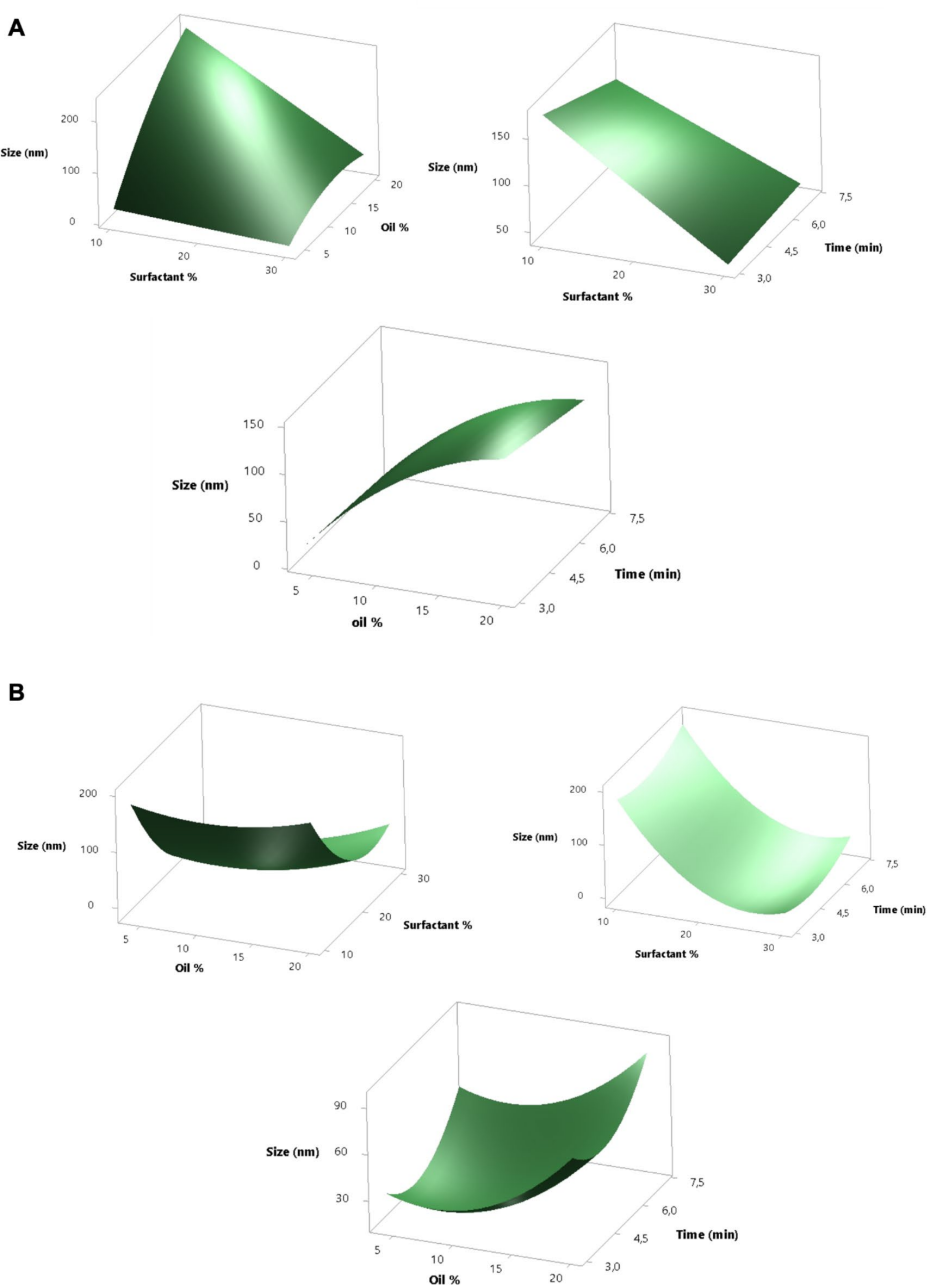
Effects of different factors of nanoemulsifying systems on the droplet size of YP (**A**) and GM (**B**) extracts. Hold values YP: Oil = 4% surfactant = 30% and time = 5 min; hold values GM: Oil = 12% surfactant = 20% and time = 5 min.

Additionally, the RSM graphs in Fig. 4A and B allow an understanding of the interaction between the factors and their influence on the response variable. In general, for both emulsions containing YP and GM extracts, the results in Fig. 4A and B show that a higher percentage of oil results in increased droplet size, whereas the opposite effect is exhibited when the surfactant concentration increases. These trends are expected considering that the obtained droplet size reflects the balance between the internal oil volume and the total surface area provided by the surfactant. In other words, a greater amount of surfactant provides a greater surface area that can stabilize the oil–water interface, which drives the formation of a greater number of smaller droplets, which has been thoroughly documented^74,76^.

The degree of droplet size uniformity can be assessed by DLS and described by the PDI, which is a dimensionless parameter ranging between 0 and 1, where 0 indicates completely uniform droplets and larger values indicate greater polydispersity. Typically, acceptable values for the PDI range between 0.05 and 0.7^77,78^. Table 2 shows that for both extracts, the PDI values of the formulations are within the acceptable range; notably, larger droplet sizes have higher PDI values and vice versa.

The zeta potential or ζ indicates the charge of the droplet surface and is presented in Table 2. These values confirm a negative surface charge for the droplets in all formulations. The values around − 40 mV are very close to the optimum for exceedingly stable cosmetic formulations^79^. Similarly, nanoemulsions based on *Sargassum* algae extract have reported zeta potentials between − 37.3 and − 50.7 mV, attributing the charge to the phenolic compounds present in the extracts that were absorbed onto the surface^80^.

Accordingly, the analysis of the nanoemulsion characteristics yielded the following final conditions for the optimal formulations. For both extracts, the sonication time was not related to the droplet size and therefore was set at 5 min. In the case of the YP extract, the lowest amount of oil (4%) and highest amount of surfactant (30%) were needed, whereas for the GM extract, a medium amount of oil (12%) and a medium amount of surfactant (20%) were required to obtain smaller droplet sizes with a predicted value of 100 nm. Table 5 summarizes the final composition and the characterization of the optimal formulations, showing a slight increase in droplet size from the expected value, probably due to the complexity of the algae extracts. However, this difference falls within the confidence interval, reaffirming the accuracy and precision of the employed model.

**Table 5:** Final characteristics of the optimized formulations for each algae extract.

Parameter	YP	GM
Size (nm)	128.5 ± 8.6	133.5 ± 4.0
PDI index	0.25 ± 0.06	0.22 ± 0.03
ζ (mV)	− 45.14 ± 0.02	− 38.6 ± 0.02
Final composition		
Extract	1%	1%
Surfactant	30%	20%
Oil	4%	12%
Water	65%	67%

The data are presented as the mean ± standard deviation (n = 3).

### Evaluation of photoprotective capacity and sun protection factor via EIS

We investigated the protective capacity of nanoemulsions containing algal extracts against UVB radiation by utilizing a previously published in vitro methodology based on EIS measurements of excised skin membranes^46^. In brief, this in vitro method utilizes the combined effect of UVB irradiation and oxidative stress to induce clear effects on the electrical properties of the skin membrane^46^. Oxidative stress is induced by adding H_2_O_2_ as a source of reactive oxygen species (ROS), while NaN_3_ is used to inhibit the antioxidative enzyme catalase, which is naturally present in the epidermis^81^. The skin membrane is mounted in a Franz cell and exposed to PBS solution containing 1 mM H_2_O_2_ and 10 mM NaN_3_ in both the donor and receptor (see Fig. 1). Thus, the EIS measurements enable the analysis of changes in the electrical resistance (∆R_mem_) and the effective capacitance (∆C_eff_) of the skin membrane induced by the combined effect of UVB irradiation and oxidative stress. Notably, exposure of these experimental conditions without any UVB protection has been shown to drastically reduce ∆R_mem_ and enhance ∆C_eff_, which is interpreted as a significant reduction in skin barrier integrity^46^. Conversely, the topical application of formulations that prevent major changes of ∆R_mem_ and ∆C_eff_ following exposure to UVB and oxidative stress signifies their protective capacity.

**Fig. 5:**
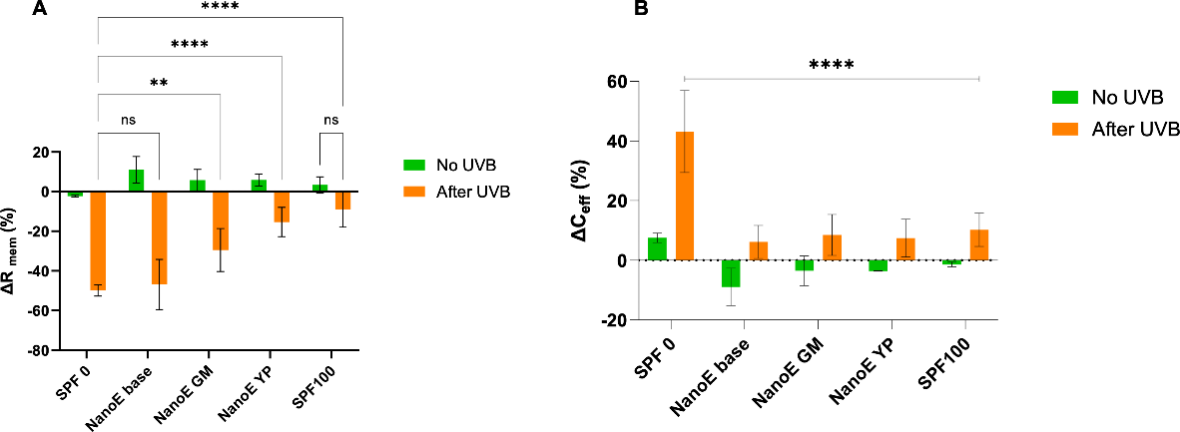
Effects of UVB radiation on the electrical properties of skin membranes and the protection capacity of nanoemulsions containing algae extracts. The data represent average values with error bars showing ± SD (n = 6 in all cases). (**A**) Photoprotective activity in terms of Δ*R*_*mem*_ (%) (**B**) Effective capacitance change Δ*C*_eff_ (%). SPF 0 corresponds to PBS solution. NanoE base correspond to the identical nanoemulsion used for NanoE GM and NanoE YP, except that no algae extract was included. SPF 100 is a commercial sunscreen formulation. In all cases, the skin membranes were exposed to 1 mM H_2_O_2_ and 10 mM NaN_3_ in both the donor and receptor compartments. Statistical significances are based a one-way ANOVA, assuming equal variances, followed by a multiple comparison test (Tukey) where **p < 0.001 and ****p < 0.0001.

Based on this methodology, we evaluated the protective capacity of the optimized nanoemulsions containing algae extracts. The results from these experiments are summarized in Fig. 5, where the bars labelled No UVB (green) represent the reference values of ∆R_*mem*_ and ∆C_*eff*_ obtained for all skin membranes without UVB exposure (i.e., the change of these parameters for each experimental condition after 5 h of a standard dose of formulation topically applied) This was done to assess whether the treatments had any significant effect on the electrical variables. These controls were conducted simultaneously with the irradiated samples and consistently revealed that the Δ*R*_mem_ was not significantly affected (less than 10%). The bars labelled after UVB (orange) represent the samples with a standard dose of the formulations topically applied, followed by 5 h UVB exposure (corresponding to a total dosage of 180 J/cm^2^). To enable relevant comparisons of the UVB protection capacity of the nanoemulsions with algae extracts, three controls were included. Firstly, SPF 0 represents UVB exposure in PBS without any topical formulation applied. Secondly, NanoE base represents application of nanoemulsion without any algae extract. Thirdly, SPF 100 represents application of a commercial sunscreen formulation with SPF 100.

The results in Fig. 5a show that the Δ*R*_*mem*_ values are consistently reduced following UVB exposure (orange bars), as compared to the reference values (green bars). However, this effect is most pronounced in the case of SPF 0 and the nanoemulsion without algal extract (i.e., NanoE base), while the nanoemulsions containing algal extracts resulted in less drastic reduction of Δ*R*_*mem*_. In fact, the nanoemulsions containing algal extracts (i.e., NanoE GM and NanoE YP) resulted in statistically significant lower reduction of Δ*R*_*mem*_ compared to the case of SPF 0, indicating that these formulations have UVB protecting capacity. Notably, no difference was found between the SPF 0 group and the NanoE base group (*p* > 0.05), which implies that the UVB protection is derived from the algae extract and not from the base ingredients of the nanoemulsion. Finally, in the case of SPF 100, the results showed no difference without UVB (i.e., no UVB, as shown in Fig. 5) and with UVB exposure (*p* > 0.05) after 5 h, demonstrating that this level of protection maintains complete membrane integrity.

As a complement to the characterization of UVB-induced alterations in Δ*R*_mem_, the EIS data were evaluated in terms of changes in the effective capacitance of the skin membranes (Δ*C*_eff_). These results are summarized in Fig. 5b, which shows a statistically significant difference between the samples corresponding to SPF 0 (i.e., no UVB protection) and those with any formulation applied on the skin (*p* < 0.0001). In conclusion, the results implies that the Δ*C*_eff_ is less suitable as parameter to grade the protecting capacity of topical formulations with varying SPF values. For example, the change of Δ*C*_eff_ corresponding to the nanoemulsions with and without algae extracts, as well as the SPF 100 formulation, show similar trends, implying that there is no correlation between Δ*C*_eff_ and the UVB protection capacity. This finding is consistent with our previous results, where no correlation between Δ*C*_eff_ and the SPF value was observed^46^. Nevertheless, a significant difference was observed between skin membranes without UVB protection (SPF 0) and those treated with SPF ranging from 10 to 70^46^. To rationalize these findings, it is relevant to first consider the components of the skin membrane that primarily contribute to the effective capacitance. In general, this parameter is related to the dielectric properties of the SC, which includes the extracellular lipid matrix, the lipid‒protein domains of the corneocytes, and any charged lipid or protein species that contribute to double-layer capacitance^64,65,82^. Thus, the results in Fig. 5b imply that treatment with 0 SPF (i.e., only PBS solution with H_2_O_2_ and NaN_3_) results in alterations in these components after UVB treatment, resulting in a drastic increase of Δ*C*_eff_ compared to the corresponding reference value Δ*C*_eff_. These findings support previous findings showing that UVB exposure causes subtle changes in the molecular mobility of SC lipids and the polypeptide backbone of keratin filaments, as well as affects SC cell cohesion and mechanical integrity^8,9^. However, with the topical application of formulations, the dielectric properties of the formulation films likely affect the measured capacitance directly. Additionally, the skin membrane’s dielectric properties may be influenced by the penetration of formulation ingredients. These factors make it challenging to distinguish the effects induced by the formulation from those caused by oxidative stress and UVB exposure. For instance, the reference values for the various formulations show negative changes in ΔC_*eff*_, suggesting a decrease in the effective dielectric constant of the skin membrane and/or the applied formulation film. This contrasts with the slight increase observed in the SPF 0 (PBS-treated) case, indicating an increase in the dielectric constant due to hydration^65^. Given this, it remains unclear why UVB-treated skin membranes with topical formulations exhibit minor positive changes in ΔC_*eff*_.

### Assessment of the SPF values of formulations containing algae extracts via EIS

**Fig. 6:**
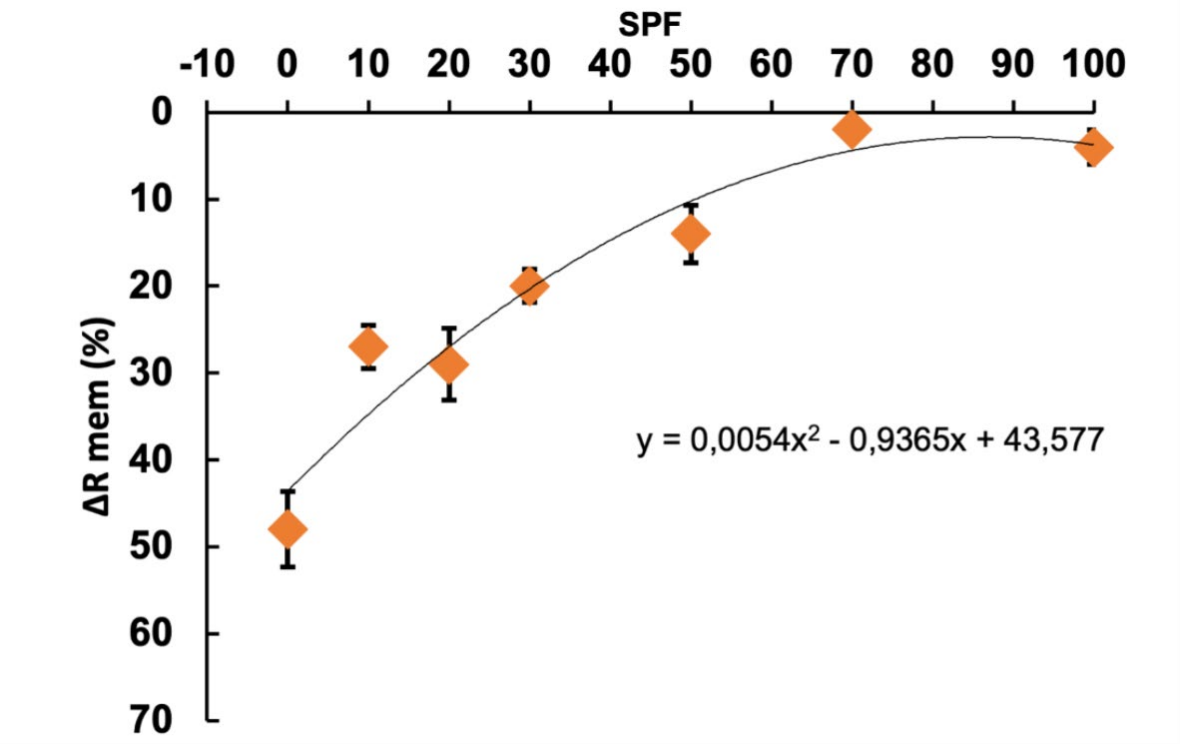
The calibration curve from our previous study^46^ included a new point from commercially available SPF 100. Polynomial correlation between UV-exposed skin membrane resistance reduction, Δ*R*_mem_%, and SPF with R^2^ = 0.932 (n = 3 ± SD).

In our previous study, we employed a similar in vitro EIS methodology and investigated different commercial sunscreen formulations with SPF values ranging from 10 to 70, showing good correlation between changes of ∆R_mem_ and SPF values^46^. In other words, ∆R_mem_ was shown to be less affected when topical formulations with higher SPF values were applied, suggesting that the methodology successfully probes the formulations capacity to maintain the skin barrier integrity and protect from damaging effects of UVB exposure. Based on these results, in combination with the results obtained in the present study with SPF 100, we analyzed the UVB-induced changes in Δ*R*_mem_ as a function of the SPF value. This evaluation is presented in Fig. 6, which shows that the UVB protection capacity has asymptotic behavior. By fitting these data with a second-order polynomial, it is possible to estimate the SPF value of the nanoemulsions containing algae extracts. Table 6 summarizes this SPF estimation.

**Table 6:** Sun protection factor estimated for the new formulations based on algae extracts.

Formulation	NanoE GM	NanoE YP
Estimated SPF	17	39

One strategy to counteract the deleterious effects of solar radiation is the use of topically applied products containing substances capable of preventing UVB/UVA rays from reaching the skin or at least reducing the number of photons that can reach tissue structures^29^. According to our findings, formulations containing algae extracts demonstrate significant UVB photoprotective ability. This supports existing research demonstrating the photoprotective properties of marine algae-derived compounds, particularly mycosporine-like amino acids (MAAs), carotenoids such as fucoxanthin, and sulfated polysaccharides like fucoidan. These compounds function through multiple mechanisms, including direct absorption of UV radiation, scavenging of ROS, inhibition of matrix metalloproteinases (MMPs) that degrade collagen, and modulation of gene expression linked to skin barrier repair and resilience. For instance, MAAs absorb UV radiation within the 310–360 nm range, acting as natural sunscreens, while carotenoids and sulfated polysaccharides mitigate UV-induced oxidative damage and inflammation, promoting skin integrity and reducing photoaging effects^83–85^. Particularly, fucoxanthin, present in both the GM and YP algae extracts, has been reported to exhibit highly effective scavenging capacity by reacting with several ROS, including peroxyl radical (ROO), hydroxyl radical (HO) and hypochlorous acid (HOCl), outperforming some benchmark antioxidants such as trolox, ascorbic acid and cysteine^86,87^. These mechanisms highlight the potential of algae extracts as sustainable and bioactive agents for mitigating the adverse effects of UVB exposure.

In our study, the YP algae extract provided better protection than the GM extract, despite fucoxanthin being less abundant in YP. This finding suggests that other compounds in YP may play a more significant role, or that the antioxidant activity of fucoxanthin could be enhanced by the nanoemulsion. Previous studies have shown that the protective effect of active ingredients can vary depending on the type of formulation used^88–90^. Another possibility is that being NanoE GM an oilier formulation than NanoE YP it could also affect the electrical properties of the skin and therefore, its photoprotective capacity. Still, the results show that GM and YP algae extracts from brown algae could be considered promising photoprotective ingredients, reinforcing previous findings for this class of species found in tropical environments^91^ and representing the first report for these species. Additionally, previous studies have shown that formulations based on natural products, particularly marine organisms, are good candidates for photoprotective formulations^92–94^. As expected, the control SPF 100 provided the greatest protection, considering that it includes multiple active ingredients (organics and inorganics) for reaching such high values. The results of this study are consistent with a previous report where this high SPF demonstrated its effectiveness in protection compared with a lower SPF^95^.

In summary, ΔR_mem_ effectively differentiates between formulations with varying SPF values, indicating that this parameter reflects the detrimental effects on skin membrane integrity caused by UVB radiation and oxidative stress. Additionally, the EIS in vitro method offers predictive capabilities while being experimentally straightforward and cost-effective. These attributes are valuable for the cosmetic industry, where simple in vitro testing for UVB protection during the early stages of product development and for evaluating new ingredients with promising photoprotective potential is essential. However, it is important to acknowledge the limitations of this methodology in replicating real-world sunscreen use conditions. Therefore, it should be noted that this methodology cannot replace the officially approved methods for determining the sun protection factor (SPF).

### Conclusion

Based on EIS and ΔRmem evaluation, this study highlights the promising potential of algae extract-based nanoemulsions as photoprotective agents against UVB radiation. The proposed nanoemulsions containing extracts from *S. cymosum* (GM) and *D. justii* (YP) demonstrated significant UVB protection. Compared with the control formulations, oil-in-water formulations containing 1% algae extract with a nanodroplet size of approximately 130 nm and low polydispersity (< 0.3) exhibited a statistically significant photoprotective capacity. Additionally, EIS has proven to be a valuable tool in photoprotection research, providing a sensitive response by directly assessing skin electrical characteristics, quantified through ΔRmem. These findings reinforce the viability of using natural marine resources for developing sustainable sun protection solutions. Future research should focus on further optimizing these formulations and exploring their long-term safety and effectiveness in real-world applications. Additionally, investigations should seek to determine whether the primarily mechanism of protection is UV absorption or ROS scavenging in maintaining skin membrane integrity. Elucidating the precise molecular mechanisms underlying the photoprotective effects of these algae-derived compounds, particularly their interaction with skin lipids and proteins would be invaluable for advancing the field and improving the efficacy of future formulations. Expanding the scope to include studies on other algae species and natural extracts may also reveal novel compounds with enhanced photoprotective properties, further enriching the range of sustainable sun protection solutions.

## Data Availability

The authors confirm that the data supporting the findings of this study are available within the article. Raw data can be shared upon request from aura.hernandez@mau.se.
